# A phase II study evaluating safety and efficacy of niraparib in patients with previously treated homologous recombination defective metastatic esophageal/gastroesophageal junction/proximal gastric adenocarcinoma

**DOI:** 10.3389/fonc.2024.1435056

**Published:** 2024-11-21

**Authors:** Ahmed Bilal Khalid, Christos Fountzilas, Heather N. Burney, Hirva Mamdani, Bryan P. Schneider, Christopher Fausel, Susan M. Perkins, Shadia Jalal

**Affiliations:** ^1^ Department of Internal Medicine, Division of Hematology/Oncology, Indiana University Melvin and Bren Simon Cancer Center, Indiana University School of Medicine, Indianapolis, IN, United States; ^2^ GI Division, Department of Medicine, Roswell Park Comprehensive Cancer Center, Buffalo, NY, United States; ^3^ Department of Biostatistics and Health Data Science, Indiana University, Indianapolis, IN, United States; ^4^ Department of Oncology, Barbara Ann Karmanos Cancer Institute, Wayne State School of Medicine, Detroit, MI, United States

**Keywords:** PARP inhibitors, PARPIs, esophageal adenocarcinoma, homologous recombination defects, niraparib

## Abstract

**Introduction:**

Esophageal adenocarcinoma (EAC) remains a devastating disease and second line treatment options in the metastatic space are limited. Homologous recombination (HR) defects have been described in EAC in up to 40% of patients. Poly (ADP-ribose) polymerase (PARP)1 and PARP2 inhibitors have shown efficacy in HR defective prostate and ovarian cancers. Here, we describe the activity of the PARP inhibitor niraparib in metastatic EAC with HR defects.

**Methods:**

In this single arm Simon two-stage Phase II study, we assessed the safety and efficacy of niraparib in patients with metastatic EAC previously treated with platinum containing chemotherapy harboring defective HR. Defective HR was defined as deleterious alterations in the following HR genes: BRCA1/2, PALB2, ATM, BARD1, BRIP1, CDK12, CHEK2, FANCA, RAD51, RAD51B, RAD51C, RAD51D, RAD54L, NBN, ARID1A and GEN1.

**Results:**

14 patients were enrolled in this study. The trial was stopped early due to slow accrual. 3 patients did not have post-treatment scans because of rapid clinical decline. The overall response rate (ORR) (95% exact CI) was 0/11 = 0% (0%, 28.49%). The disease control rate (DCR) (95% exact CI) was 2/11 = 18.2% (2.3%, 51.8%). The median PFS was 1.8 months (95% CI = 1.0-3.7). The median OS for evaluable patients was 6.6 months (95% CI =2.7-11.4) and 5.7 months for all patients (95% CI =2.7-10.1). The most common adverse events seen were anemia, fatigue, and thrombocytopenia.

**Conclusion:**

In patients with metastatic EAC, single agent niraparib as second line therapy is not an effective option.

## Introduction

Nearly 1/2 million patients are diagnosed with esophageal cancer annually and outcomes remain poor as most patients present with locally advanced or metastatic disease (1). The standard of care in the first line setting of HER2 negative metastatic esophageal adenocarcinoma (EAC) is a platinum-based regimen in combination with a fluoropyrimidine and a checkpoint inhibitor in patients with positive Programmed Ligand 1 (PDL1) expression (2). Second line options are limited and targeted therapies for specific genomic subsets are restricted to HER2 positive esophageal cancer (3).

Homologous recombination (HR) defects have been described in EAC and TCGA analysis have shown abnormalities in HR genes in up to 40% of patients (4). HR plays a role in genome integrity maintenance through repair of DNA double stranded breaks (DSBs). Poly (ADP-ribose) polymerase (PARP)1 and PARP2 are DNA-binding enzymes that play a crucial role in DNA repair. Tumors with HR repair deficiency (HRD) caused by loss-of-function mutations in BRCA1 or BRCA2 genes display increased sensitivity to PARP inhibition and multiple studies in HR defective prostate (5) and ovarian cancer (6) have shown activity of PARP inhibitors. The synthetic lethality of PARP inhibitors in BRCA1 and BRCA2-deficient cells possibly extends to tumors with deficiency in other HR-related and DNA damage signaling genes (CDK12, PALB2, ATM and CHK) and is known as the BRCAness phenotype of a tumor (7).

In this study, we assessed the activity of the PARP inhibitor niraparib in metastatic EAC with HR defects.

## Methods

This was a single arm Simon two-stage Phase II study evaluating the safety and efficacy of niraparib as second line therapy in patients with metastatic esophageal/gastroesophageal junction (GEJ)/proximal gastric adenocarcinoma harboring defective HR previously treated with platinum containing chemotherapy. Patients with disease progression during the first 2 months of chemotherapy were excluded. The null hypothesis was that the ORR was 10% and the alternative hypothesis was that the ORR was 25%. The Type I (alpha) error was 5% and power was 80%. In stage I of the two-stage design, 18 patients were to be treated, and if 2 or fewer responses were observed, the trial would be stopped early for futility. 25 additional patients would be enrolled if the trial advanced to stage II. If fewer than 7 patients responded in stage II phase, the drug would not be considered worthy of further study in this patient population. Defective HR was defined through previously characterized deleterious alterations in HR genes including loss-of-function/pathogenic/or likely pathogenic as specified per the following databases: Clinvar, OncoKB, or BRCAExchange. Next-generation sequencing was used to evaluate genetic aberrancies in candidate genes through the HR pathway. Patients were deemed to be eligible if they had a deleterious alteration in one of the following: BRCA1/2, PALB2, ATM, BARD1, BRIP1, CDK12, CHEK2, FANCA, RAD51, RAD51B, RAD51C, RAD51D, RAD54L, NBN, ARID1A and GEN1. Patients with germline mutations in HR genes specified were also allowed.

The trial was conducted by the Big Ten Cancer Research Consortium and enrolled patients at 2 sites. IRB approvals at all sites and informed consents were required from all patients. For patients weighing ≥ 77 kg and a baseline platelet count > 150, niraparib was started at 300 mg daily while patients who weighed < 77 kg or had a platelet count < 150 were started at niraparib 200 mg daily. Niraparib was administered orally daily for 28 days and continued until disease progression, unacceptable toxicity, death, or withdrawal of consent. The primary endpoint, overall response rate (ORR), was defined as the percentage of patients who reached complete response (CR) or partial response (PR) by RECIST 1.1. The secondary endpoint disease control rate (DCR) was defined as the percentage of evaluable patients with stable disease (SD) for 8 weeks, or PR or CR according to RECIST 1.1. The secondary endpoint of progression-free survival (PFS) was assessed using the Kaplan-Meier method. PFS was determined from start date of treatment to date of progression for patients who progressed or date of death for patients who died without progressing. The secondary endpoint of safety was assessed by summarizing all treatment-related adverse events (trAEs) as defined by the NCI Common Terminology Criteria for Adverse Events (NCI CTCAE) v5 among all patients who received at least one dose of niraparib. Overall survival (OS) was determined from start date of treatment to date of death for patients who died. Efficacy endpoints (ORR, DCR, and PFS) were assessed for patients who were considered evaluable. Evaluable patients were those who received at least one dose of niraparib and either underwent at least one post-baseline assessment or died before any evaluation. All analyses were performed using SAS Version 9.4.

## Results

Between September 2019 and October 2022, 14 patients were enrolled in the study. The trial was stopped early due to slow accrual. Baseline characteristics of study participants are shown in **Table 1**.

**Table 1:** Baseline patient characteristics.

Characteristic	n (%)
Patients On Study	14
Site	
Indiana University Melvin and Bren Simon Comprehensive Cancer Center	11 (78.6)
Roswell Park Cancer Institute	3 (21.4)
Gender	
Female	1 (7.1)
Male	13 (92.9)
Race	
White	13 (92.9)
Black or African American	1 (7.1)
Ethnicity	
Non-Hispanic	14 (100)
ECOG Performance Status	
0	3 (21.4)
1	11 (78.6)
Median Age, years (Min-Max): 59 (25-74)

### Treatment administered (n=14)

The median number of cycles received on trial was 2 (range, 1-5). 50% of the patients (n=7) were able to receive the study regimen without dose modifications. Six patients required dose modifications because of adverse events while one patient had a dose modification due to non-adherence.

### Efficacy

Response rates are available for 11 patients as 3 patients did not have post-baseline scans because of rapid clinical decline. The ORR (95% exact CI) was 0/11 = 0% (0%, 28.49%). The DCR (95% exact CI) was 2/11 = 18.2% (2.3%, 51.8%). Both patients who had stable disease at 8 weeks harbored an ARID1A mutation. One additional patient had a best response of SD but did not maintain SD for 8 weeks. The median PFS was 1.8 months (95% confidence interval = 1.0-3.7). The median OS for evaluable patients was 6.6 months (95% confidence interval =2.7-11.4) and the median OS for all patients was 5.7 months (95% confidence interval =2.7-10.1).

### Safety/toxicity

Treatment-related adverse events are listed in **Table 2**. Seven grade 3-4 trAEs were observed. Treatment was discontinued in 3 patients due to adverse events. There were no treatment-related deaths. The most common adverse events noted were anemia, fatigue, and low platelet counts with most being grade 1 or grade 2.

**Table 2:** Treatment-related adverse events.

CTCAE Term	Any Grade	Grade ≥ 3
Anemia	7	1
Fatigue	7	1
Platelet count decreased	7	0
Nausea	6	1
Headache	5	0
White blood cell decreased	5	1
Anorexia	3	0
Constipation	3	0
Weight loss	3	0
Abdominal pain	2	0
Diarrhea	2	0
Dizziness	2	0
Dyspepsia	2	0
Hypocalcemia	2	0
Lymphocyte count decreased	2	1
Neutrophil count decreased	2	1
Vomiting	2	1
Arthralgia	1	0
Aspartate aminotransferase increased	1	0
Cough	1	0
Dry mouth	1	0
Dry skin	1	0
Dysgeusia	1	0
Dyspnea	1	0
Hypercalcemia	1	0
Hyponatremia	1	0
Sinus tachycardia	1	0

### Genetic details

The individual genomic alterations in patients are listed in **Table 3**.

**Table 3:** Individual genetic details.

Patient ID	Germline mutation (blood)	Gene(s) with Mutation Present
325-1001	No	NBN
325-1003	No	ARID1A
325-1004	Yes	ARID1A
325-1005	Yes	BRIP1; CHEK2
325-1006	No	BRCA2
325-1007	No	ARID1A
325-1008	No	BRCA1; BRCA2
325-1009	No	ARID1A
325-1010	No	ATM
325-1011	No	ATM; BARD1
325-1012	No	ARID1A; ATM; PALB2
325-1013	Yes	RAD51
325-1014	Yes	BRCA2
325-1015	Yes	CHEK2

## Discussion

Our clinical trial was stopped early due to slow accrual. There was lack of activity of single agent niraparib in the first 14 accrued patients. While our sample size was small, our data suggests that single agent niraparib as second line therapy is not an effective option in the treatment of HR defective EAC. The toxicity profile of niraparib was similar to prior studies (6).

HRD impairs the ability of a cell to effectively repair DNA DSBs. While initial clinical studies in prostate cancer suggested that a number of HR mutations predict sensitivity to PARP inhibitors, subsequent studies showed that the benefit was primarily seen in patients with BRCA mutations (5). There remains a dire need to identify biomarkers that predict sensitivity to PARP inhibitors. Loss of heterozygosity (LOH) has been shown to be another potential predictor of PARP inhibitor sensitivity but at the time of our study, there wasn’t a CLIA certified assay to measure LOH (8).

Despite finding success in ovarian and prostate cancers, the utility of PARP inhibition in EAC remains unclear. The phase III GOLD trial failed to show a significant improvement in OS with the addition of Olaparib to paclitaxel in patients with previously treated advanced gastric cancer in both the overall study population and in those with ATM deficiency (9). The role of PARP inhibitors in GI cancers is currently limited to a small subset of pancreatic cancers with germline BRCA1 mutations and no progression 16 weeks post platinum (10). It is possible only a small subset of EAC tumors benefit from PARP inhibition. Further characterization of these potential subsets *in vitro* is needed prior to conducting clinical trials with single agent PARP inhibitors in EAC.

## Data Availability

The original contributions presented in the study are included in the article/supplementary material. Further inquiries can be directed to the corresponding author.
